# Supplementary figures and images for: Precise genomic mapping of 5-hydroxymethylcytosine via covalent tether-directed sequencing
Source: PLoS Biol. 2020 Apr 10;18(4):e3000684. doi: 10.1371/journal.pbio.3000684 (PMC7176277; doi:10.1371/journal.pbio.3000684)

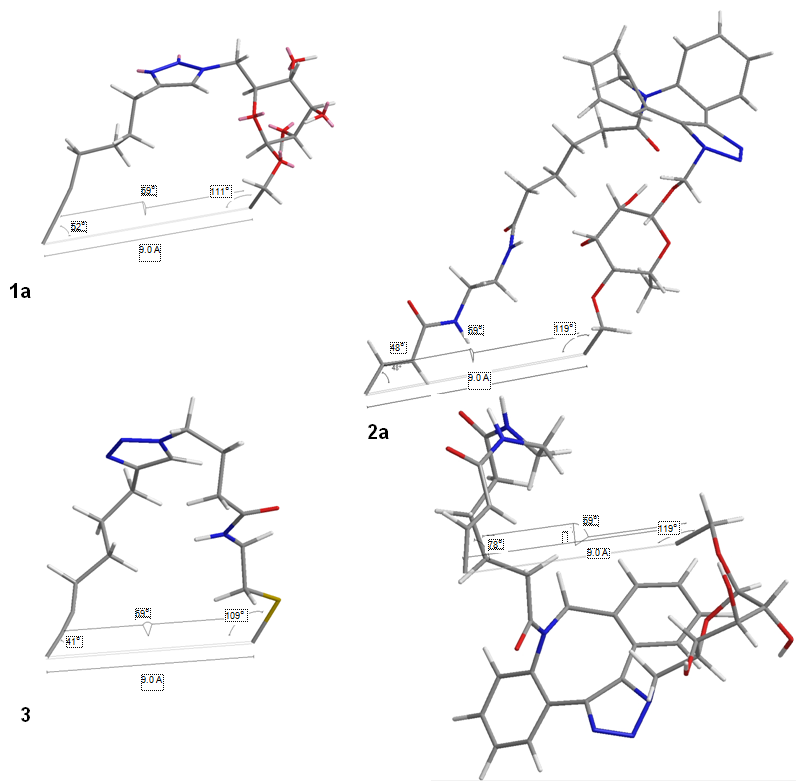

Supplement: S1 Fig — Linker conformations were determined by ChemBio3D Ultra MM2 energy minimization using the target values derived from the coordinates of dT4 and dT6 nucleotides in the template strand of the KOD polymerase–DNA complex (PDB: 5omf) as follows: C5–C5 distance = 8.99 Å; C5-methyl bond angles for dT6 (corresponds to T2 in the tethered ODN) and dT4 (corresponds to 5hmC) = 58.8° and 124.4°, respectively; dihedral angle (dT4–dT6 helical twist) = 59.8°. Actual refined values are shown in fine print. 5hmC, 5-hydroxymethylcytosine; ODN, oligodeoxyribonucleotide; PDB, Protein Data Bank. (TIF) [file pbio.3000684.s001.tif]

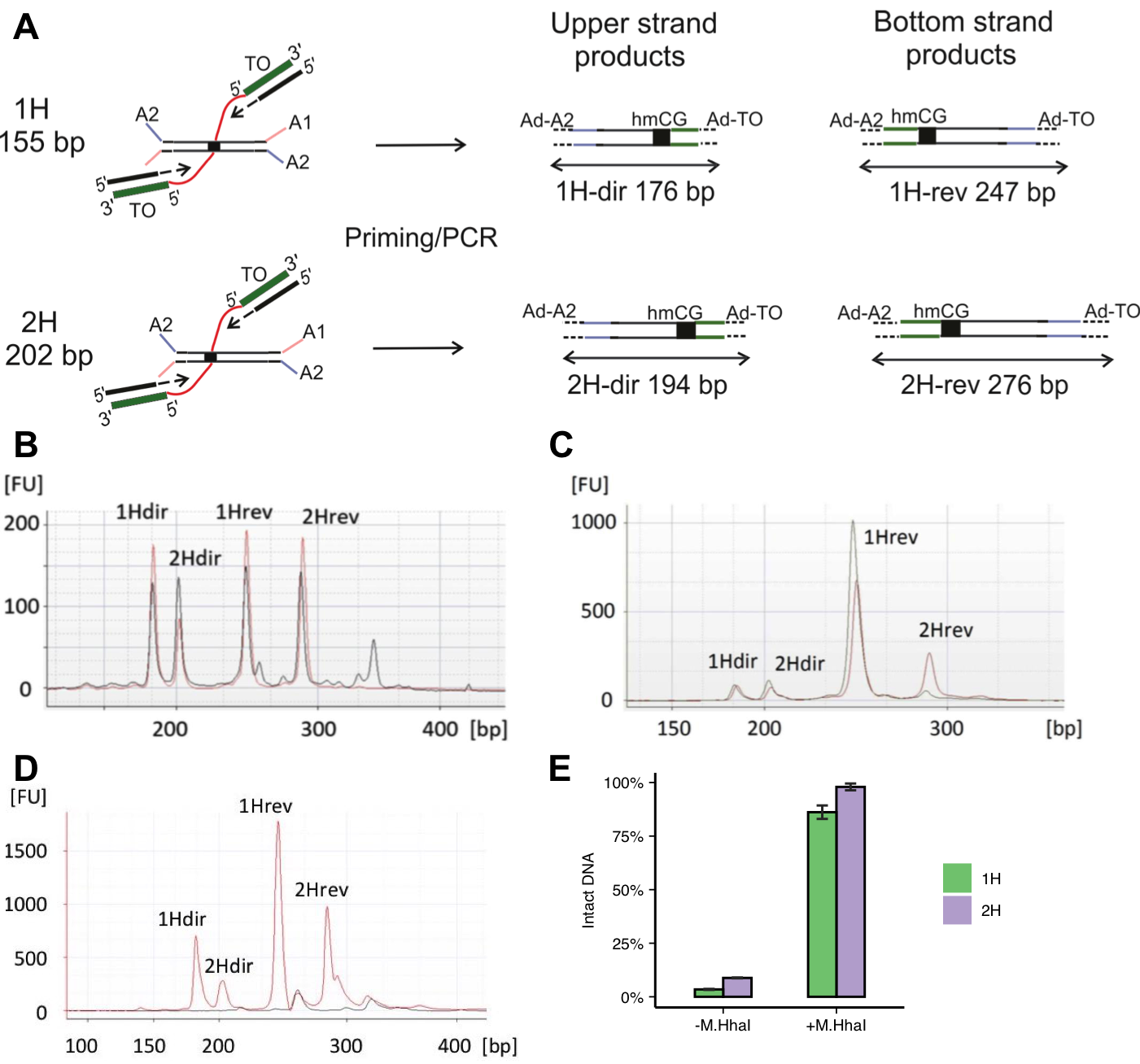

Supplement: S2 Fig — (A) Schematic view shows four priming products generated from the two model DNA fragments, 1H and 2H. Theoretical sizes of the four specific hmTOP-seq and uTOP-seq products (including 135-bp adapters) are as follows: 1H-dir 176 bp, 2H-dir 194 bp, 1H-rev 247 bp, and 2H-rev 276 bp. (B) Agilent Bioanalyzer profiles of hmTOP-seq priming products obtained from 1H and 2H model DNA fragments, each containing a single 5hmC, followed by derivatization with cysteamine (black line) or the azide group (red line). For each type of derivatization, different number of PCR cycles was required to detect comparable amounts of four products (20 cycles for azide- and 25 cycles for cysteamine-derivatization). (C) Comparison of hmTOP-seq and uTOP-seq [25] in the 1H/2H model DNA system. Bioanalyzer profiles of the products obtained from 1H and 2H model DNA fragments each containing a single 5hmC or an unmodified CG processed through the hmTOP-seq (red line) or uTOP-seq procedure (black line). In both cases, the corresponding workflow generates four specific products with similar efficiencies (15 cycles of PCR were used). Azide-labeling of unmethylated CG sites in the model DNA fragments was performed as described [25]. (D) Agilent Bioanalyzer profiles of hmTOP-seq priming products obtained from 1H/2H, followed by copper-free (black line) or Cu(I)-catalyzed (red line) click conjugation to DNA oligonucleotide. In both cases, 25 cycles of PCR were used. (E) Assessment of the M.HhaI-directed hydroxymethylation efficiency on two model DNA fragments. After incubation of 1H/2H model DNA fragments with M.HhaI in the presence of formaldehyde, the DNA fragments were cleaved with Hin6I restriction endonuclease and the amount of uncleaved DNA was evaluated by qPCR with the respective primer pairs (1H-dir/1H-rev or 2H-dir/2H-rev; see “Validation of hmTOP-seq in a model DNA fragment system” in Materials and methods). The data underlying this section are included in S2 Data. 5hmC, 5-hydroxymethylcytosin [file pbio.3000684.s002.tiff]

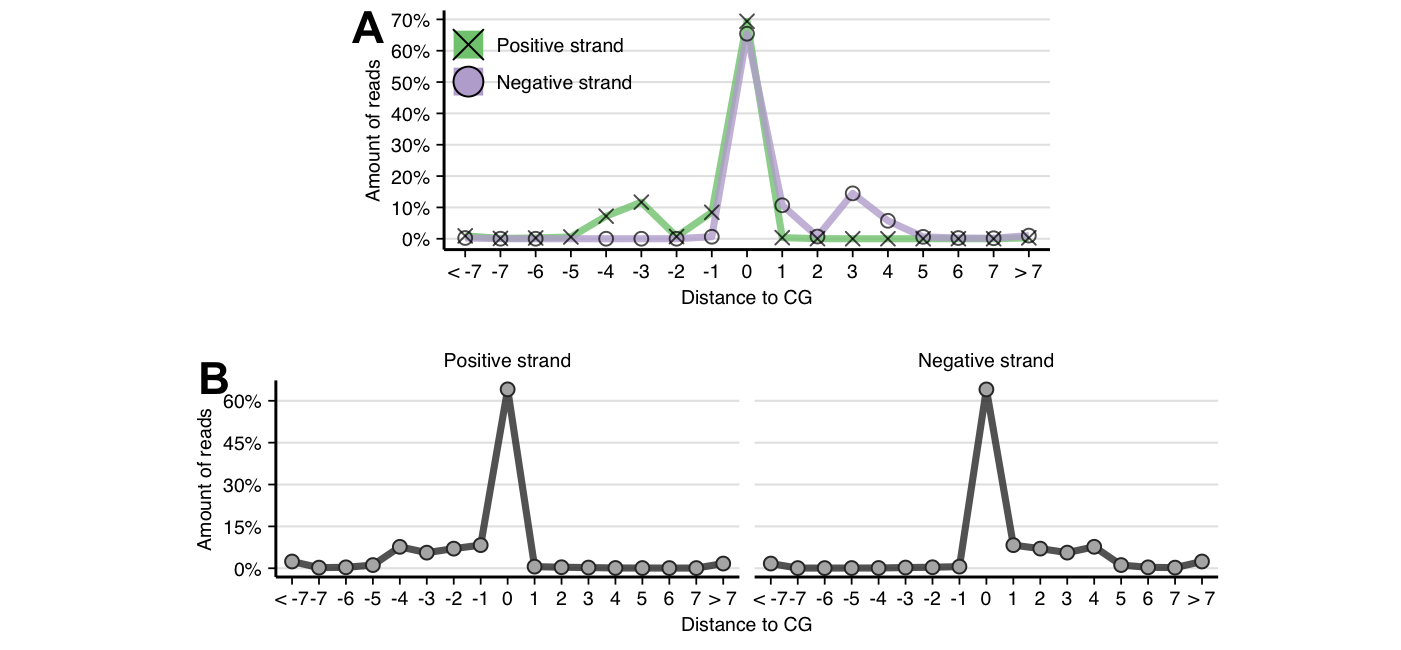

Supplement: S3 Fig — Distance distribution of read start positions from (A) a nearest GCCG or (B) a CG site in the hmTOP-seq library of pre-hydroxymethylated lambda DNA (2.5% 5hmC at GCGC sites) and mESCs, respectively. The data underlying this figure are included in S2 Data. 5hmC, 5-hydroxymethylcytosine; hmTOP-seq, 5hmC-specific tethered oligonucleotide–primed sequencing; mESC, mouse embryonic stem cell. (TIFF) [file pbio.3000684.s003.tiff]

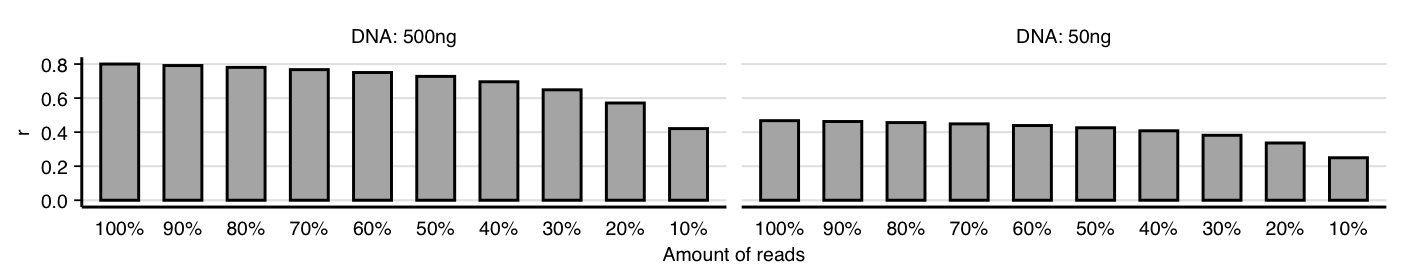

Supplement: S4 Fig — Sequencing reads were sampled from 500-ng and 50-ng input DNA hmTOP-seq libraries, respectively. The data underlying this section are included in S2 Data. hmTOP-seq, 5hmC-specific tethered oligonucleotide–primed sequencing. (TIFF) [file pbio.3000684.s004.tiff]

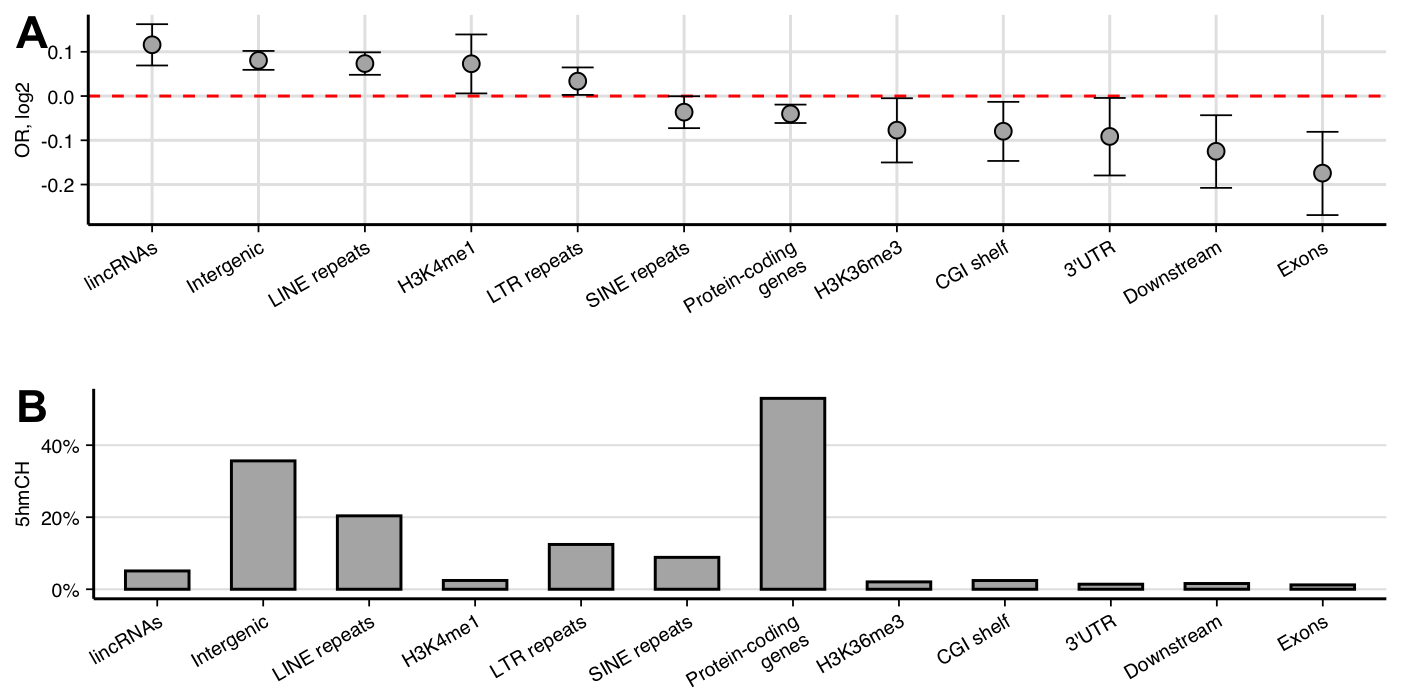

Supplement: S5 Fig — Odds ratio (Fisher’s test) for enrichment (A) or distribution (B) of 76,665 5hmCHs detected in mESCs across various genomic features. All enrichments have p < 0.05. The data underlying this section are included in S2 Data. 5hmCH, hydroxymethylated CH site; hmTOP-seq, 5hmC-specific tethered oligonucleotide–primed sequencing; mESC, mouse embryonic stem cell. (TIFF) [file pbio.3000684.s005.tiff]

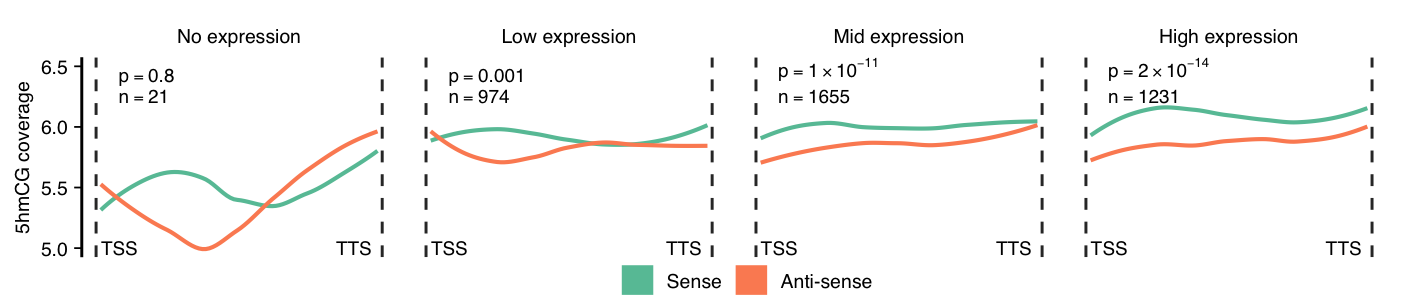

Supplement: S6 Fig — Distribution of 5hmCGs in 50-ng input DNA hmTOP-seq libraries across the sense and the antisense strands of genes grouped according to their expression level. Numbers of genes in each group and p-values for the modification difference between the strands are shown above each graph. hmTOP-seq, 5hmC-specific tethered oligonucleotide–primed sequencing; mESC, mouse embryonic stem cell. (TIFF) [file pbio.3000684.s006.tiff]

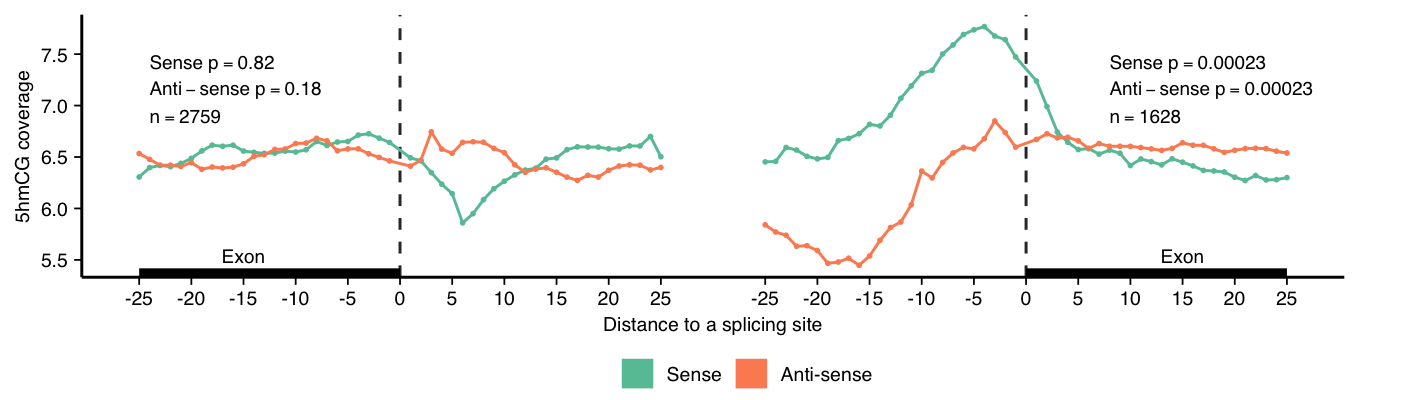

Supplement: S7 Fig — Distribution of 5hmCGs in 50-ng input DNA hmTOP-seq libraries at both sides of the exon-intron boundary is presented for the sense and the antisense strands. The x-axis shows the distance (nt) of CGs from boundary. p-Values indicate a difference in coverage between exonic and intronic side of the boundary for the first 25 nt. The data underlying this section are included in S2 Data. 5hmCG, hydroxymethylated CG site; hmTOP-seq, 5hmC-specific tethered oligonucleotide–primed sequencing; nt, nucleotide. (TIFF) [file pbio.3000684.s007.tiff]
