# Supplementary material for: Precise genomic mapping of 5-hydroxymethylcytosine via covalent tether-directed sequencing
Source: PLoS Biol. 2020 Apr 10;18(4):e3000684. doi: 10.1371/journal.pbio.3000684 (PMC7176277; doi:10.1371/journal.pbio.3000684)
Supplement: Attachment — Submitted filename: Rebuttal letter.pdf [file pbio.3000684.s012.pdf]

We wish to thank the reviewers for their thorough comments, which helped us to improve the revised manuscript.

Point-by-point responses to the Reviewers' comments

Rev. #1:

Minor comments:

1. Would the authors mention the shortcomings of this method? In general, all protocols have some issues that need to be performed carefully for optimal results.

As suggested by the reviewer, we inserted a description on the limitations of the method in Discussion section on pp. 11-12.

2. Similarly, a stepwise working protocol i.e. easy to follow must be included in supplementary section for reproducibility of the protocol.

As suggested by the reviewer, we provided a more detailed protocol of the hmTOP-seq in Materials and Methods section on pp. 15-17 (or pp.15-16 in the clean version of the manuscript) to ensure its reproducibility. We believe that a lengthy stepwise protocol does not fit with the journal format.

Rev. #2:

Major comments:

1. The initial analysis of the technique on model DNA fragments is weak and very badly explained. The authors must re-explain what is happening in Figure S2, as it is not clear. This is vital as it underpins their whole technology.

We thank the reviewer for their comments. The hmTOP-seq strategy was presented in Figure 1D. For added clarity, we included a detailed scheme in Figure S2A in order to better illustrate the origin and DNA structure of the four products generated by hmTOP-seq from the two model DNA fragments.

It is important for them to sequence the amplified products to find out what percentage actually prime at the 5hmC site verses the neighbouring sites.

We have performed sequencing analysis of the primed DNA products from bacteriophage lambda model DNA (48.5 kbp, 215 hmC target sites) and believe that this experiment, presented in Figure 2 A-C and Figure S3A, offers a much better illustration on how hmTOP-seq works at various CG sequence contexts, as compared to the initial experiment involving just two short DNA fragments. The experiment clearly shows that the majority of reads identify exact CG positions and a minor fraction of reads start just a few nt away from a CG site.

Additionally, the use of ‘high’, ‘medium’, and ‘low’ in Table S1 is unacceptable. The authors must quantify their findings. For instance, in the text they mention the DBCO addition ‘failed to generate any substantial amounts of the priming’, but in Table S1 they mark it as ‘low’.

As recommended, we replaced the statements in the Table S1 with the priming and coupling efficiency values. The coupling efficiency is presented as a percentage of model DNA fragment coupled to ODN, and the priming efficiency now is presented as a threshold cycle number (lower number corresponds with higher efficiency) of the 1H-dir/1H-rev priming products (the structures of 1H-dir/1H-rev are shown in Fig S2A) measured by qPCR using 2.5 ng of the ODN-coupled 1H model DNA fragment and the respective primer pairs (EP and 1H-dir or EP and 1H-rev).

2. The analysis of the technique on the bacteriophage was also lacking. How does formaldehyde and bacteriophage genomic DNA form 5hmC? Did they analyse the DNA to confirm the yield of this reaction? They authors seem to assume 100% yield.

Production of model DNA containing 5hmC at GCGC sites was carried out exploiting the HhaI methyltransferase-directed coupling of formaldehyde to the C5-position of the target cytosine residues, our previously discovered atypical reactivity of DNA cytosine-5 methyltransferases. Using a well established protocol (originally reported in Liutkeviciute et al, 2009 and 2011, and also briefly described here in Methods on p.13 (or p.12)) we consistently achieve nearly complete hydroxymethylation of GCGC sites in various DNA substrates. We added an explanation on this on p.5 and included an additional graph as Figure S2E. The hydroxymethylated lambda DNA was used for mixing with unmethylated lambda DNA to prepare the indicated theoretical percentages of 5hmC. This experiment shows that the hmTOP-seq coverage linearly increases with higher 5hmC levels. We believe that knowing the exact absolute values of each point in the curve is not so important for a proof-of-principle experiment like the one presented here, but surely a more elaborate calibration would be required for precise quantitative determination of hmC in biological samples.

Why was analysis only considered at GCGC sites? The authors should have again mapped the priming sites to see if this matches the analysis of the synthetic DNA?

This is exactly what we did - we mapped all hmTOP-seq reads obtained from the model lambda DNA and found that the vast majority of them start exactly at or the immediate vicinity (0-4 nt) of a GCGC site (215 GCGC sites in total). We also observed a very similar read distribution in the experiment with mouse ESCs (Fig 2C and Fig S3B). We added a new Figure S3 showing the distribution and percentage of reads starting at a particular distance to GCGC sites in lambda DNA and mouse ESCs.

Additionally, in Fig 2B, why was a curved line used as a trend line, this looks linear through the medians?

We agree with the reviewer that linear regression can better fit the data presented in Figure 2B and modified the figure according to the reviewer's recommendation.

3. Regarding the analysis of the mESCs data, the quantification of mapped reads around CG sites is vague. In the text it says 93% 'at or around CGs', why can't this be quantified to % at each position relative to the 5hmC. By the Fig 2C this looks to be ~65% at the 5hmC site. This should then be compared to the data from the model DNA and bacteriophage mapping, which was not conducted. I think I understand, but the authors should really explain why their method can analyse asymmetric 5hmC so well (I believe because the direction of priming has to be 5' to 3'). It would be good to make a figure for this.

The reviewer is correct by saying that ~65% of reads start exactly at CG sites. As mentioned above, the reads starting within the distance -4/+4 to a CG sites identify the same CG, thus we summed up these reads and this resulted in the estimate that 93% of reads start at or in a near proximity to CG sites. The percentage of reads starting at each particular distance can be seen in Figure 2C and Figure S3B. As the main finding is that the majority of reads start directly at CG sites, the authors feel that providing the exact percentages at each position to CG sites does not add much to clarity of the text.

We added the read distribution analysis (Figure S3) of lambda DNA. Additionally, as recommended by the reviewer, we included in Figure S2A the schematic view representing the structure and origin of the products formed by the priming reaction from the two model DNA fragments.

4. A lot of the time is unclear if the mESC data is being analysed with only the 500 ng library, or combined with either or both of the 50 and 5 ng libraries. This needs to be clear at all times. How many replicates of each library size were prepared and analysed? The pearson correlation coefficient is good for the 500 ng library, but poor for the 50 ng library and no correlation is seen for the 5 ng library. This indicates that they require at least 500 ng input DNA for good coverage of 5hmC (even if less of the library can be sequenced following this). This goes against one of the main advantages the authors are claiming. Additionally, how good is it to then use data from the 50 and 500 ng libraries together?

The 5, 50, or 500 ng libraries were analyzed separately and never combined. We have indicated throughout the text and in the Figure legends the library type used for each experiment.

All hmTOP-seq libraries were prepared in two technical replicates. Sequencing statistics is presented in Table S2. For better clarity, we included a statement about the replicates of lambda and mESCs libraries on p.5

We agree with the reviewer that correlation with lower input libraries is not as good as with 500 ng. However, the correlation increases with calculation of h-density. The choice of input DNA amount for hmTOP-seq analysis depends on the purpose of the experiment.

While 50 ng input could still be used for analysis of relative modification levels of CGs at single-nucleotide resolution, we recommend that with lower input libraries 5hmC density (h-density) should be better employed for regional comparison of 5hmC levels. We included this recommendation on pp.11-12.

5. The authors analyse 5hmC in non-CG context with their method. However, has it been previously shown that the b-GT is active outside of the CG context? If not, the authors should quantify this activity in synthetic DNA to confirm their method is actually functional in these contexts.

The specificity of the BGT enzyme was examined and described in these manuscripts:

1. Josse, J. & Kornberg, A. Glucosylation of deoxyribonucleic acid. III. alpha and beta-glucosyl transferases from T4-infected *Escherichia coli*. *J. Biol. Chem.* **237**, 1968–1976 (1962).

2. Lariviere, L. & Morera, S. Structural evidence of a passive base-flipping mechanism for beta-glucosyltransferase. *J. Biol. Chem.* **279**, 34715–34720 (2004).

3. Song C-X, Szulwach KE, Fu Y, Dai Q, Yi C, Li X, et al. Selective chemical labeling reveals the genome-wide distribution of 5-hydroxymethylcytosine. *Nat Biotechnol* **29**, 68–72 (2011).

As authors claim, the enzyme does not have any sequence specificity and thus modifies all 5hmC residues independent of the sequence context. Therefore, it can be used for examining cytosine hydroxymethylation levels outside the CG context.

Other comments:

6. More citations are required where reference 1 is, to highlight the amount of work that has been carried out into quantifying the levels of 5hmC across multiple tissue types.

We expanded the reference list with additional articles (references 1-11) describing 5hmC analysis in mouse and human tissues, as recommended by the reviewer.

7. 5hmC is only mentioned as an intermediate in the demethylation pathway, please include the extra details that demonstrate it may be an epigenetic mark in its own right.

We modified the introduction section by mentioning the role of 5hmC as an independent epigenetic regulator on pp. 1-2 and included additional references on this (references 1-6).

8. The authors mention that high sequencing depths is ‘prohibitively expensive’ (page 2). I think this is unfair, as many groups do sequence to high depths. Prohibitively is not the correct term.

We removed this term and rephrased the sentence.

9. As the authors use the ‘TOP-seq’ method also, they need to explain how this works.

The uTOP-seq (TOP-seq of unmodified CGs) method was presented in Stasevskij et al, (2017), therefore, we provided the link to this study without detailed explanation of the method itself, except for the statement that the method detects unmodified CG sites. The strategy of uTOP-seq is exactly the same as that of hmTOP-seq, except for the first covalent labeling step. Additionally, enrichment of the primed products on streptavidin beads was added to the hmTOP-seq protocol, which is not necessary for the analysis of abundant DNA modifications or unmodified CG fraction.

10. The ‘h-density’ is not explained at all, and I do not know what this term refers to. Please explain why they have used this, and what the ‘180 bp window’ is. Does this remove the single base resolution advantage?

The term “h-density” refers to average coverage of 5hmCG sites within 180 bp windows normalized to regional CG density. In the legend of Figure 2D, we included a sentence on how the h-density is calculated.

Yes, we agree with the reviewer that h-density does not provide single CG resolution. However, h-density is a useful metrics for comparison of regional 5hmC changes among multiple samples or tissue types, especially when analysis of individual CGs is not very instructive due to shallow coverage. In principle, the size of the analysis window can be varied depending on the purpose. For better compatibility with uCG-TOP-seq data, we chose the window of 180 bp which has been refined in Stasevskij et al (2017).

11. At the end of page 8 the authors say they compare ‘hydroxymethylation and general cytosine modification levels’ using hmTOP and TOP. How was this done as the authors haven’t demonstrated that TOP-seq can show where modified cytosines are present, just where non-modified cytosine is present. I don't see how they can call a modified cytosine from the negative of TOP-seq, as their method is not truly quantifiable.

Indeed, uCG-TOP-seq measures unmodified CGs. In our previous paper on the TOP-seq technology (ref. 25), we have demonstrated that the TOP-seq signal inversely correlates to the regional CG modification levels, and can well be used to profile genomic DNA methylation.

12. The authors mention reference 18 in passing. However, this is an incredibly important literature reference, as they perform almost the same method described within. This should be described in more detail. From reading reference 18, it does seem this paper has a major advantage of closer to single-base resolution, but this needs to be discussed in more detail.

The method mentioned in reference 18 (now ref. 27) has appeared just before submission of our article, and thus it could not have had any effect on the motivation or

execution of our work up to the very final moments. From our perspective, it presents a version of the hmTOP-seq method with one of the least successful choices of the chemical linkage (see DBCO linkages in Table S1; although, to be exact, the chemical structure of the DBCO linkage used was not even disclosed in that publication). Eventually, when we turned to comparing the two methods, we were unable to do this even theoretically due to numerous inconsistencies of their sequencing data deposited in the repository. For some replicates, the number of regions between the strands differ drastically (e.g. for replicate 1 there are 289,628 regions in the minus strand and only 27,399 regions in the plus strand; for replicate 5 there are 68,275 regions in the minus strand and only 10,685 in the plus strand). Also, the number of regions differ a lot between replicates. There are 130,736 regions reported on average per replicate, however some replicates have a very low number of regions (e.g. replicate 3 had only 2,650 regions). Finally, the files that should have contained information from all 11 merged replicates turn out to be corrupted. The file for the plus strand "GSE127906_48ng_11rep_merge.plus.FDR0.05.corrected.bed" contains only peak coordinate information, but lacked coverage values and, therefore, is completely useless.

Altogether, were were unable to confirm/reproduce their reported findings and statements, nor could we perform any meaningful comparison of the deposited data. Our main conclusion is that bulky DBCO linkages (like the one used in ref. 18 (the new ref. 27)) afford rather inefficient and inaccurate (within 20 nt) priming of the polymerases as compared to those proposed in our work, which leads to inherently lower resolution of the method (so-called “near CpG resolution”). At this point, we believe that no further comments on the recently published method are necessary.

13. Why was LNA and phosphorothioate modifications used on the priming strand (page 14)?

Generally, LNA modifications are known to increase the stability of DNA duplexes. In our study, LNAs were used for increasing the annealing temperature of the primer and thus the specificity of the priming step. Phosphorothioate modifications were included at the 3’ end of the priming oligonucleotide in order to protect it from the 3’-5’ exonuclease activity of Pfu polymerase.

Rev. 3:

I have following criticisms, which need to be addressed before publication:

1. It is important to benchmark performance of hmTOP-seq in ability to detect 5hmC sites with different modification frequency. Authors could use TAB-seq data to select sites with different degrees of 5hmC and examine correlation with hmTOP-seq coverage.

We thank the reviewer for the positive feedback and this important comment. We included an additional graph into Figure 2, as a subsection 2E, which demonstrates a good

agreement between hmTOP-seq coverage and 5hmC percentage estimated by TAB-seq. Also, we added the sentence describing this relationship on p. 6 (or p.7).

2. What are the sources of the inefficiency of hmTOP-seq - is it labelling of 5hmCs or DNA polymerase priming from non-complimentary oligonucleotide?

The overall efficiency is the product of the following three steps. Previous studies and this work demonstrated that the BGT enzyme typically modifies 5hmC residues in DNA with high efficiency. The Click reactions also worked quite well except for the cases of 6-azido glucose and bulky DBCO group (Table S1). In the TOP step, the Pfu non-conventional polymerase priming depends on proper stacking of the tagged nucleotide at the end of the tethered DNA duplex, which may require certain structural and physical conditions to be met, and some of which (length and structure of the tethering linker, its attachment position to TO) has been further optimized in the current work.

3. Authors state - "Using six hmTOP-seq control libraries we observed 55,025 false-positive sites". I was not able to find what are the cut-offs for false positives and what criteria were used to define them.

In all control libraries, we were able to identify only 55,025 non-CG sites with at least one read per cytosine. Due to the small number of identified sites with the lowest coverage cut-off and their poor overlap between control libraries we defined those sites as background false-positive cytosines and removed them from further non-CG analysis. The previous sentences on p.6-7 were corrected accordingly.

4. When discussing strand bias instead of term "opposite" strand, "template" strand should be used.

We thank the reviewer for this suggestion on the usage of terms. As the term “template” strand is used for describing the priming mechanism from the Tethered oligonucleotide duplex, we decided to use the terms “sense” and “anti-sense” when describing the coding and opposite strands, respectively, to avoid confusion.
