# Supplementary material for: Precise genomic mapping of 5-hydroxymethylcytosine via covalent tether-directed sequencing
Source: PLoS Biol. 2020 Apr 10;18(4):e3000684. doi: 10.1371/journal.pbio.3000684 (PMC7176277; doi:10.1371/journal.pbio.3000684)
Supplement: Attachment — Submitted filename: Rebuttal letter.pdf [file pbio.3000684.s013.pdf]

Point-by-point response to the Reviewer 2 comments

The authors have substantially improved their manuscript. I only have two further comments:
1) Regarding the trend line in Fig 2B. After plotting a linear regression, this also does not look as though it fits. Potentially it is linear from 0-20, but then it plateaus. I didn't mean for the authors to just put a straight line on the graph. They should actually use some statistics to identify the trend.

Yes indeed, one can visually identify a linear trend followed by a progressively slower increase. To account for this bimodality, we applied binomial approximation, which contains both a linear term and a second order term corresponding to possible saturation. Using raw read count data (to avoid any possible mathematical skewing) we found a very good agreement (as compared to linear fit) with $Y=99.5+15.7X-0.240X^2$ (adjusted R^2 =0.9266; p=0.000107). We added the fitting information in the figure legend, and an amendment in the main text (p.5), and changed information accordingly in the Methods section (p.16).

2) The authors mention input quantity as an improvement over bisulfite sequencing in the introduction "demands sizeable amounts of input DNA" and the discussion "hmTOP-seq allowed the construction of 5hmC maps with several hundred-fold less starting DNA amounts than for WGBS". The authors method does have some distinct advantages over WGBS commonly, however input quantity is not one of them and 'hundred-fold less' is grossly overstating even to the conventional bisulfite sequencing in input quantities (0.5-5 µg DNA) (DOI10.1186/s13059-018-1408-2). At this reference, you can also find links to papers where WGBS has been performed on a few hundred cells to even single cells. In the authors manuscript 5 ng could not be used for single base resolution.

WGBS is a well-established technology and has been exploited in many experimental modalities for different purposes that use different amounts of input DNA and give a variety of output data in terms of accuracy, resolution, coverage depth etc. Therefore, a direct comparison with a newly developed technique is always problematic. Although input DNA amounts for WGBS is in the range of 0.5-5 µg DNA, many WGBS studies used high input quantities. The input DNA in hmTOP-seq single-base resolution analysis is 50-500 ng which is less than in most routine WGBS studies. To avoid misunderstanding, we removed the term 'hundred-fold less' and amended the text on p. 12 as follows: “The non-destructive nature of hmTOP-seq allowed the construction of 5hmC maps with less starting DNA amounts than for routine WGBS”.
